# Recycling Ophthalmic Lens Wastewater in a Circular Economy Context: A Case Study with Microalgae Integration

**DOI:** 10.3390/ma17010075

**Published:** 2023-12-22

**Authors:** Telma Encarnação, Nadia Nicolau, Pedro Ramos, Elsa Silvestre, Artur Mateus, Tomás Archer de Carvalho, Florindo Gaspar, Anabela Massano, Sara Biscaia, Ricardo A. E. Castro, Bernardo A. Nogueira, Poonam Singh, Diana Pacheco, Tatiana Patrício, Rui Fausto, Abílio J. F. N. Sobral

**Affiliations:** 1 Coimbra Chemistry Centre-Institute of Molecular Sciences (CQC-IMS), Department of Chemistry, University of Coimbra, 3004-535 Coimbra, Portugal; rcastro@ff.uc.pt (R.A.E.C.); bernardoalbuquerquenogueira@gmail.com (B.A.N.); diana.m.pacheco@ipleiria.pt (D.P.); rfausto@ci.uc.pt (R.F.); asobral@ci.uc.pt (A.J.F.N.S.); 2 Centre for Rapid and Sustainable Product Development (CDRSP), Polytechnic Institute of Leiria, 2430-028 Marinha Grande, Portugal; artur.mateus@ipleiria.pt (A.M.); tomas.carvalho@ipleiria.pt (T.A.d.C.); florindo.gaspar@ipleiria.pt (F.G.); anabela.p.massano@ipleiria.pt (A.M.); sara.biscaia@ipleiria.pt (S.B.); tatiana.patricio@ipleiria.pt (T.P.); 3 PTScience, Avenida do Atlântico, N 16, Office 5.07, Parque das Nações, 1990-019 Lisboa, Portugal; nadia.nicolau@ptscience.pt (N.N.); pedro.ramos@ptscience.pt (P.R.); elsa.silvestre@ptscience.pt (E.S.); poonam.singh@ptscience.pt (P.S.); 4 Opticentro, 2460-071 Alcobaça, Portugal; 5 Faculty of Sciences and Letters, Department of Physics, Istanbul Kultur University, Ataköy Campus, Bakirköy, Istanbul 34156, Turkey

**Keywords:** sustainability, biobased products, biopolymers, microalgae, bioremediation, circular economy, ophthalmic lenses, recycling

## Abstract

Water pollution poses a global threat to ecosystems and human health and is driven by the presence of various contaminants in wastewater, including nano- and microplastics. Despite the magnitude of this problem, the majority of global wastewater is released untreated into water bodies. To combat this issue, a multi-strategy approach is needed. This study explores a circular economy-based solution for treating emerging pollutants, particularly wastewater from ophthalmic spectacle lens production. Our approach integrates solid waste materials into polymeric and cement matrices while also utilising wastewater for microalgae cultivation. This innovative strategy focuses on biomass generation and economic valorisation. By adopting a circular economy model, we aim to transform environmental pollutants from wastewater into valuable organic products. A key component of our approach is the utilisation of microalgae, specifically *Nannochloropsis* sp., known for its high lipid content and resilience. This microalgae species serves as a promising biobased feedstock, supporting the production of innovative biobased products, such as biopolymers, for ophthalmic lens manufacturing. Our interdisciplinary approach combines microalgae technology, analytical chemistry, cement production, and polymer processing to develop a sustainable circular economy model that not only addresses environmental concerns, but also offers economic benefits. This study underscores the potential of harnessing high-value products from waste streams and underscores the importance of circular economy principles in tackling pollution and resource challenges.

## 1. Introduction

The circular economy (CE) concept has gained popularity worldwide in recent years and has gradually emerged as a solution to growing concerns about climate change, chemical pollution, the depletion of natural resources, the collapse of ecosystems, and health implications. Due to increased concerns and environmental regulations and programmes, the concept has gained increased attention from academics and companies; under the CE concept, countries are implementing funding programmes and policies [1,2] as an alternative to the currently prevailing linear economic model. The linear economic model requires an infinite supply of natural resources to feed production and an environment with unlimited capacity to deal with waste and pollution [3].

The CE concept has been labelled a “theoretical dream” and not an “implementable reality” [4]. The controversy around the concept is most related to its definition and implementation. According to a previous study, an analysis of more than 100 different definitions, CE can be defined as “an economic system that replaces the ‘end-of-life’ concept with reducing, alternatively reusing, recycling, and recovering materials in production/distribution and consumption processes” [5]. Among the many definitions and controversies of CE, it is accepted that, overall, it implies an economic model based on sustainable development; environmental quality, economic prosperity and social equity are the three pillars of sustainable development. Aligned with the CE concept, there has been increasing research and a set of regulations regarding pollutants released into water bodies [5]. 

Wastewater, like any other waste, is a matter of concern for the environment in the context of sustainable development. Recycling wastewater and treating the toxic elements released have become urgent for the better management of a green planet. In general, wastewater treatment involves a combination of physical, chemical, and/or biological processes. These processes segregate colloids, organic matter, nutrients, and soluble contaminants from effluents. The characteristics of wastewater discharge determine the method to be applied; however, due to technological and economic reasons, only a few of these methods can be used on an industrial scale. 

**Figure 1:**
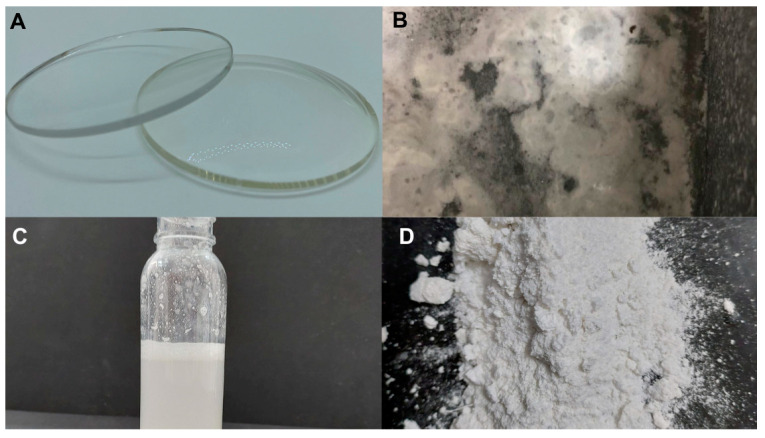
Ophthalmic spectacle lens wastewater: (**A**): lens before griding; (**B**): wastewater with lens grinding residues; (**C**): optic industry wastewater supernatant; (**D**): solids from ophthalmic spectacle lens wastewater.

One specific example, among many other unsustainably managed wastes, is derived from the ophthalmic spectacle lens industry (Figure 1). Ophthalmic lenses follow a unique distribution system dictated by the need to fill prescriptions. Producers are engaged in preparing ophthalmic lenses (Figure 1A), as prescribed, for final distribution by optical stores. This stage, in which lenses produced by a factory need to be shaped into the frames, will be our focus. During this process, around 10 L of water per lens is needed. This process is rarely carried out in a closed circuit, which means that this waste goes untreated into sewage. The waste is composed of humid waste (Figure 1B,C) that ends up in landfills, and the untreated water with micro- and nanoplastics, along with many other organic pollutants, are discharged into sewage and eventually released into the environment. In addition to the polymeric matrix, an ophthalmic lens is a complex system composed of many other different kinds of additives and multicoatings, some of which are considered emerging pollutants and endocrine-disrupting chemicals, such as BPA, benzene, toluene, and heavy metals [6]. According to market reports, in 2019, 703.2 million pieces were sold [7]. Considering that part of this material is discarded untreated, potentially causing environmental issues, it is urgent to find possible solutions that could solve or at least reduce its impact on the environment. 

Among the different wastewater treatment technologies available, bioremediation using microalgae could be a potential alternative to integrate with conventional water treatment processes [8,9]. Microalgae are photosynthetic unicellular organisms used for many decades for the bioremediation of nutrients such as nitrates and phosphates, and more recently for the removal of emerging pollutants, such as pharmaceuticals and pesticides [10,11,12]. Beyond the bioremediation of pollutants, microalgae also have the potential to become a significant biobased source of feedstock for numerous products and applications [13,14]. Bioplastics, biofertilisers, and biochemicals represent only a fraction of the vast potential that these micro-organisms may provide. Microalgae possess the ability to fix CO_2_ and utilise organic and inorganic carbon, nitrogen, and phosphorus through photosynthesis, effectively releasing O_2_ into the atmosphere. This not only contributes to reducing carbon footprints by capturing CO_2_, but also minimises the emissions of nitrogen oxides (NOx) and sulphur oxides (SOx). Furthermore, the symbiotic relationships between microalgae and other microbes enable the breakdown of complex pollutants in wastewater. The bioactive compounds produced as byproducts of microalgae cultivation also have diverse industrial applications, contributing to economic growth and sustainability. 

Microalgae can grow under photoautotrophic, heterotrophic, and mixotrophic conditions, amongst others, which make it viable for them to bloom under most conditions. One interesting and extremely resilient species of microalgae is the *Nannochloropsis* genus. *Nannochloropsis* sp., the species used in this study, is an invaluable resource as a biobased feedstock due to its exceptional characteristics, notably its high oleaginous content, which may reach circa 80% dry weight [15]. This unique attribute of *Nannochloropsis*, i.e., its remarkably high oil content, positions it as an ideal candidate for biobased feedstock. 

Considering the environmental crisis and the imperative for more sustainable practices, it becomes evident that urgent viable solutions are required. Apart from awareness interventions, legislation, and waste policies, technical solutions are also needed. In this study, we propose a potential solution that combines recycling, recovery, and the use of alternative feedstocks such as microalgae. Minimising, utilising, recovering, recycling, and reusing resources from waste are addressed through the cultivation of microalgae in this article in the context of the circular economy concept. Therefore, the objective of this research work is to present a multidisciplinary case study in which the concept of a circular economy is tested and its implementation proposed: ophthalmic spectacle lens wastewater was used; the solid waste was incorporated as a filler in two different matrices; the aqueous waste was remediated using microalgae; and a value-added product obtained from the resulting biomass was successfully proposed.

To the best of our knowledge, this is the first approach to address ophthalmic spectacle lens waste using the circular economy concept.

## 2. Materials and Methods

### 2.1. Wastewater, Organism, and Culture Medium

The ophthalmic spectacle lens wastewater (WW) (Figure 1) was obtained from Opticentro^®^ (Alcobaça, Portugal) with a custom waste retrieval system applied on a water jet lens cutter. 

*Nannochloropsis* sp. was acquired from Varicon Aqua Solution (Malvern, UK). The culture was grown for 6 days in 2 L f/2 medium [16]. The culture was aerated by bubbling atmospheric air at a rate of 300 cm^3^ min^−1^, and a temperature of 25 ± 2 °C was maintained. 

### 2.2. Experimental Design

**Figure 2:**
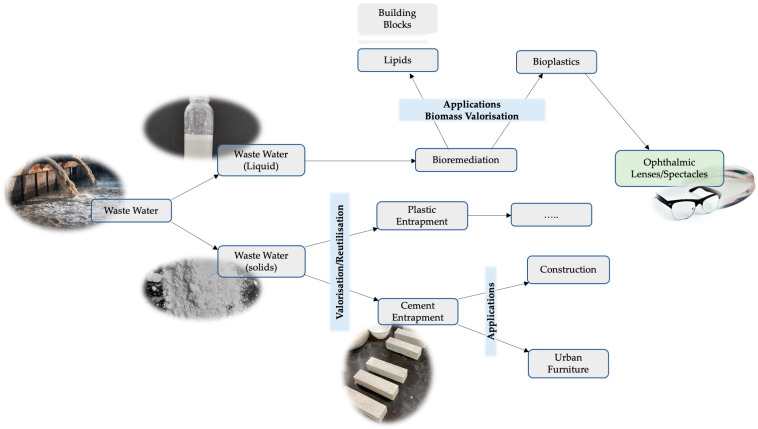
Experimental design illustrating the sequence of the experiments: Wastewater contains a mixture of solid pollutants, including nanoplastic and microplastic residues, and liquid components. After the separation of solid pollutants from the liquid portion of the wastewater, the separated solid pollutants are incorporated into cement (cement entrapment). This results in the creation of a cement composite that entraps the solid pollutants. These cement mortars can potentially offer a sustainable solution for the development of urban furniture. Additionally, the solids can also be entrapped in plastics. The liquid portion of the separated wastewater is employed in microalgae cultures for bioremediation. Subsequently, the biomass generated from the bioremediation process has several applications. The biomass can be used to produce lipids, which serve as valuable building blocks for a range of products, thereby contributing to the circular economy. After the extraction of lipids, the remaining biomass can be used to produce lactic acid, which, in turn, can be utilised to produce polylactic acid (PLA). This PLA has advanced applications, such as in the manufacturing of ophthalmic lenses, demonstrating the high-value outcomes of our approach.

For an easier understanding of the experimental design, Figure 2 illustrates the sequence of the experiments and a crucial aspect of our experiment, depicting the procedure for wastewater treatment and the various innovative applications derived from it. 

### 2.3. Ophthalmic Spectacle Lens Wastewater and Solid Waste Profile

The industrial wastewater profile was determined using Fourier-transform infrared (FTIR-ATR) and Raman spectroscopies.

The chemical composition of the samples was analysed via FTIR spectroscopy, using an Alpha FT-IR spectrometer (Bruker, Kontich, Belgium) and Opus Software. All assays were performed at room temperature, in a spectral range of 400–4500 cm^−1^, with a resolution of 2 cm^−1^ in a total of 64 scans.

The Raman spectra of the spectacle lens solid waste profile were collected on a Horiba LabRam HR Evolution Raman equipment, with a solid-state laser source excitation at 785 nm, focused through a 50× magnification long-working-distance (LWD) lens. The spectra were collected in the wavenumber range of 50–1800 cm^−1^, at an average of 1000 individual collections of 10 s each.

### 2.4. Ophthalmic Spectacle Lens Solid Waste for Polymer-Based Composites

The wastewater-suspended solids were incorporated as a filler material into polymer-based composites. The polymer-based (low-density polyethylene) plastic waste solid (WW) composites (WW/LDPE) were obtained using a co-rotating twin screw extruder. The chemical composition of the polymer-based composites was analysed via FTIR spectroscopy.

All spectra were obtained in stopped-flow and flow-through modes at a nominal 4 cm^−1^ resolution by co-adding 25 scans in the first 10 min and 125 scans in the remaining time of spectra acquisition. The background spectra were obtained using the empty ATR crystal covered with the appropriate tested polymer as a reference.

### 2.5. Ophthalmic Spectacle Lens Solid Waste for Cementitious Mortards 

**Figure 3:**
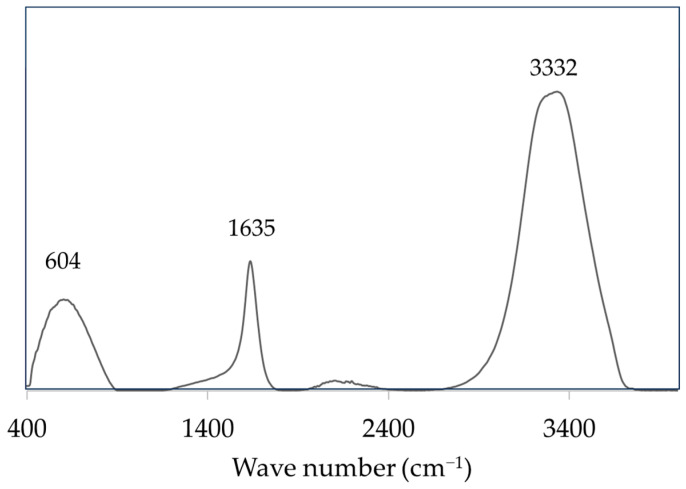
FTIR-ATR spectrum of the aqueous fraction of the ophthalmic spectacle lens wastewater.

The wastewater-suspended solids were incorporated as a filler material into the formulation of cementitious mortars. The incorporation of ophthalmic spectacle lens waste as a filler material into the formulation of cementitious mortars (Figure 3) was assessed in terms of its impact on mechanical performance, namely on the compressive strength achieved at 7 days. The mortars were composed of Portland cement (CEM I 42.5 R), natural, washed, and classified sand (<1 mm), and limestone powder or plastic waste dust used as fillers.

A fixed weight ratio of cement/aggregate (sand + filler) of 1:2 was used. The proportion of sand and plastic waste filler used was decided based on particle packing optimisation, using an optimisation curve. Optimisation curves are theoretical particle size distributions (PSDs) that lead to an optimisation of the mixture’s overall particle packing density. In other words, the mixing proportions of the various materials are such that the mix PSD is as close as possible to the selected optimisation curve, thus maximising the packing density [17].

In the present study, Fuller’s curve was used as the optimisation curve:

(1) $$P\left(d\right)=\sqrt{\frac{d}{d_{max}}}$$

Here, *P*(*d*) is Fuller’s PSD cumulative function (the optimisation curve), *d* is the distribution function variable and represents particle size, and *d_max_* is a parameter of the distribution function and represents the maximum particle size in the considered mixture.

The process of determining the volumetric proportion of each material is then based on adjusting the calculated mix curve (which, of course, depends on the proportions of the materials) to the optimisation curve. This can be achieved, e.g., using a graphical method [13] in which, starting from the higher-grade material (sand, in this case), the ideal volumetric proportion of each material is defined. In the present study, since the cement/aggregate ratio was fixed, determining the proportion of sand allowed us to define the mix composition.

Based on the optimal mix proportions obtained, a series of ophthalmic spectacle lens waste-containing mortar specimens were prepared (PWM), and the results of the compressive strength at 7 days were compared with those from a reference cementitious mortar (RM), using limestone as filler instead of ophthalmic spectacle lens waste.

A usual mortar-mixing procedure was carried out: Cement and water were mixed in an automatic mixing machine (Controls, 65-L0502) for about 30 s at a low mixing speed. Then, the sand and filler materials were added, and the mixing process was continued for another 90 s at high speed. After mixing, and 15 min after the initial addition of water to the cement, a flow table test was carried out [18]. Then, 40 × 40 × 160 mm moulds were filled with fresh mortar and left to cure under ambient conditions (temperature around 22 °C and relative humidity around 60%) for 1 day. Thereafter, the specimens were demoulded and added to water at room temperature for the following 6 days. The same mixing procedure and curing conditions were used for both the reference mortar and the plastic-waste-containing mortars [18].

After 7 days of curing, 6 specimens of each mortar were tested in a universal testing machine (Instron, 4505) and their compressive strength was determined.

### 2.6. Ophthalmic Spectacle Lens Wastewater for Microalgae Cultivation

After the suspended solids were separated and then incorporated into the formulation of the cementitious mortars and polymer-based composites, the aqueous fraction of the ophthalmic spectacle lens wastewater was used to cultivate microalgae.

Four different simultaneous sets of experiments were conducted at 25 ± 2 °C under light with an irradiance level of 100 μmol m^−2^ s^−1^, with a 16:8 photoperiod, and were run for 8 days. The cultures were aerated by bubbling atmospheric air at a rate of 300 cm^3^ min^−1^. 

Millipore water was added when needed to ensure constant volume due to water loss through evaporation. 

The experiments differed in the contents of the culture solutions, where “NN” was a culture composed of f/2 culture medium and *Nannochloropsis* sp.; “NNWW” contained f/2 culture medium, *Nannochloropsis* sp., and wastewater; “WW” contained f/2 culture and wastewater; and finally, “WW” contained only wastewater. A quantity of 500 mL of *Nannochloropsis* sp. and f/2 culture medium was filtrated and washed, and the cells were then added to the f/2 culture medium and wastewater experiments (NN and NNWW). In the samples containing f/2 culture medium and wastewater, namely, NNWW and WW, the contents of the f/2 culture medium were dissolved in 100 mL of wastewater to maintain a total volume of 100 mL, and in NN, the medium was dissolved in distilled water. A volume of 5 mL of each sample was collected every 24 h and the samples were frozen at −18 °C until analysis. To compensate for the volume of the collected sample, 5 mL of the respective solution was added. A mathematical correction was applied to compensate for the dilution effects, according to a method described previously [11]. The control group is represented by the standard medium for *Nannochloropsis* sp., referred to as F2 medium (NN). This medium did not contain the pollutants present in wastewater.

The cellular density was determined daily, using the *Neubauer* chamber in an optical microscope. Each experiment was carried out in triplicate.

The microalgal biomass was filtered and dried for 48 h at 37 °C to screen the main compounds produced by *Nannochloropsis* sp. when grown in industrial wastewater and under ideal circumstances. The biomass was then subjected to FTIR-ATR analysis, and spectra were recorded with the OPUS program (Bruker, MA, USA). All spectra are the average of two independent observations ranging from 400 to 4500 cm^−1^ and taken over 64 scans at a resolution of 2 cm^−1^.

### 2.7. Valorisation of Microalgal Byproducts 

The microalgae biomass generated in the bioremediation process can be converted into feedstock for biobased products for advanced applications. One such application includes ophthalmic lenses made from polylactic acid (PLA). After bioremediation using microalgae, and the maximisation of lipids and their extraction, it is possible to obtain feedstock for producing biopolymers; the lipids can be used as a feedstock to produce various chemicals [15]. For convenience, PLA was acquired from NatureWorks^®^ (Minneapolis, MN, USA).

The feasibility of PLA for applications in advanced optical products was evaluated through thermal and optical analyses using Differential Scanning Calorimetry (DSC), polarised light thermo-microscopy (PLTM), and refractive index and Abbe number measurements.

### 2.8. Statistical Analysis 

A single-factor ANOVA analysis was used to determine the statistical significance of each treatment in terms of the microalgae population viability. The experiments were performed in triplicate and data were subjected to one-way analysis of variance (ANOVA). Multiple comparisons were performed via a Tukey test. Statistical significance was set at *p* < 0.05 using SPSS (SPSS Inc, Chicago, IL, USA). The density data are presented as means ± standard deviation, Microsoft Excel^®^ (Microsoft Corp., Redmond, WA, USA) was used to perform statistical analysis, and a *p*-value < 0.05 indicates statistical significance.

## 3. Results

### 3.1. Ophthalmic Spectacle Lens Wastewater and Solid Waste Profile

The FTIR-ATR spectra of the aqueous fraction of the ophthalmic spectacle lens wastewater (Figure 3) revealed three main broad peaks, specifically at 604 cm^−1^, 1635 cm^−1^, and 3332 cm^−1^. 

**Figure 4:**
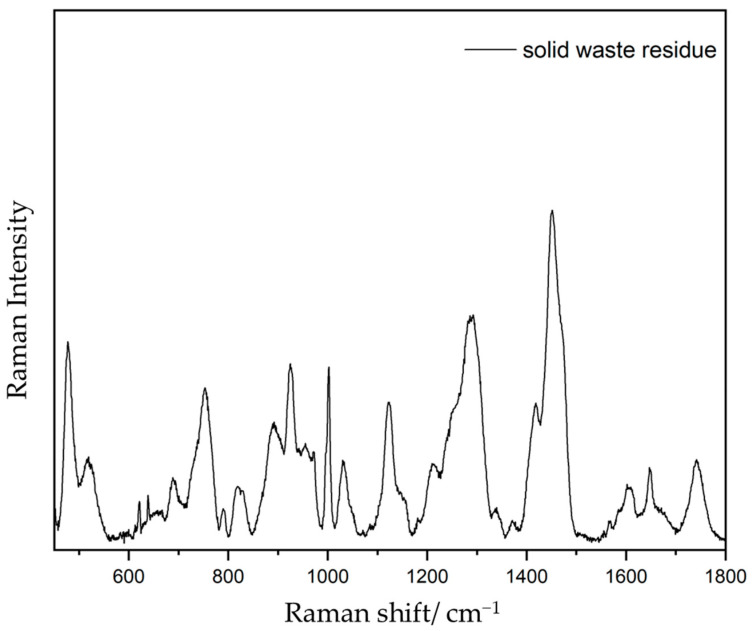
Raman spectrum of solid waste residue fraction of the ophthalmic spectacle lens wastewater.

The Raman spectroscopy analyses of the industrial wastewater profile (Figure 4) revealed the presence of different compounds, specifically through the presence of peaks at 1605 cm^−1^, 1451 cm^−1^, and 1288 cm^−1^ and an intense band at 1002 cm^−1^.

### 3.2. Ophthalmic Spectacle Lens Solid Waste Valorisation

**Figure 5:**
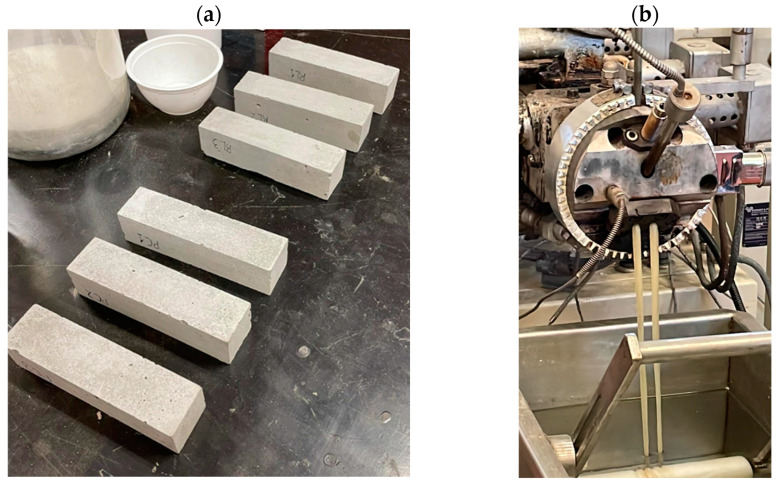
(**a**) Plastic waste cementitious mortars and (**b**) polymer composites.

The wastewater-suspended solids were valorised through their incorporation as a filler material into cementitious mortars (Figure 5a) and polymer-based composites (Figure 5b). 

#### 3.2.1. Polymer-Based Composites (WW/LDPE)

The physical structural study of polymeric chains and their deformation and aggregation provides defining elements for the application and development of polymeric materials.

**Figure 6:**
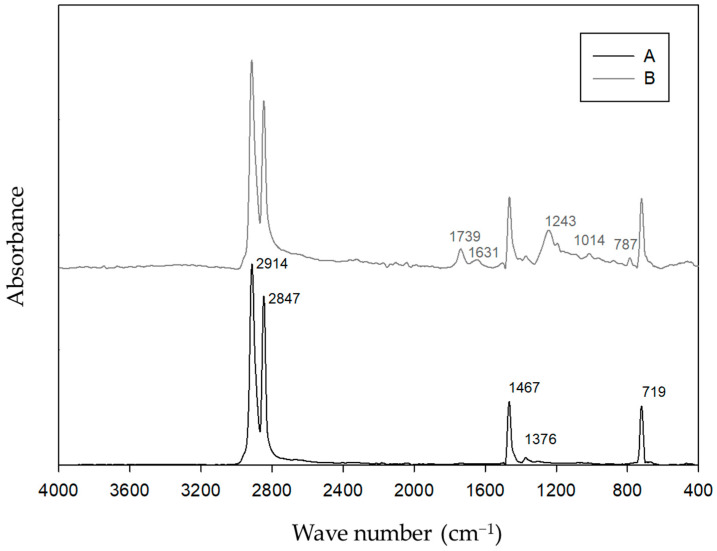
FTIR spectra of LDPE (A) and LDPE/LENS (B) composites.

The LDPE and LDPE composite spectra (Figure 6) showed characteristic strong important peaks at 2915 and 2847 cm^−1^. These spectra also revealed 1466 and 1376 cm^−1^ bands, as well as a peak at 719 cm^−1^.

#### 3.2.2. Cementitious Mortars 

**Figure 7:**
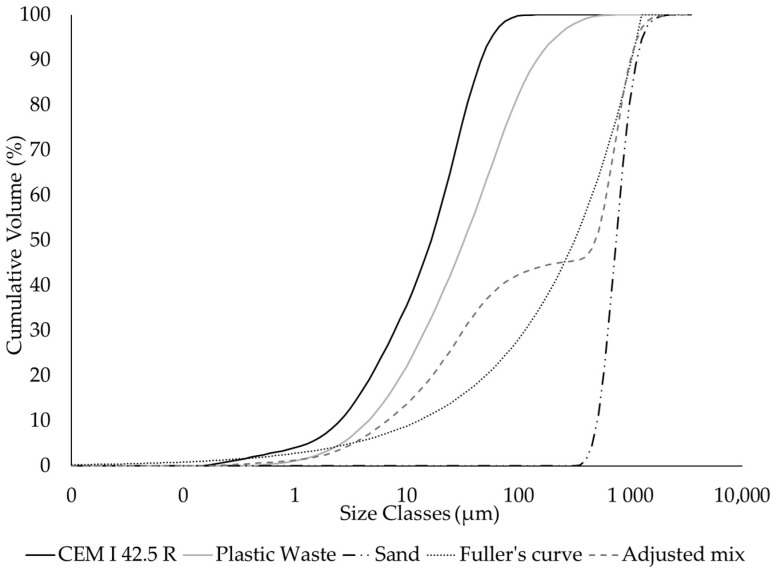
Measured cumulative PSD curves for cement CEM I 42.5 R, plastic waste dust, and sand: Fuller’s optimisation curve and adjusted mix PSD curve.

The PSD curves for the CEM I 42.5 R cement, plastic waste dust, and sand used in the preparation of cementitious mortars are shown in Figure 7. Fuller’s optimisation curve and the adjusted mix curve are also presented.

For this case, according to the above-mentioned methodology, the optimal mix composition was defined as shown in Table 1. The weight proportions were calculated based on the density of each material and are also presented below.

The density figures for the cement and sand were obtained from the respective suppliers. For the case of the plastic waste dust, since the composition was not known, the density of the material was estimated via a displacement method, using water as the displaced fluid. In this method, given that the plastic waste is insoluble in water, the volume of a weighted amount of plastic waste was estimated by adding it to a volumetric flask that was then filled to the mark with deionised water at 20 °C. The volume of water needed to fill up the flask was measured with a calibrated burette and the volume of the weighted amount of plastic waste was obtained by difference. The obtained density of 1.30 × 10^3^ kg m^−3^ was well in line with a hypothetical mix of mid- and high-index plastics (density of 1.1–1.3 and 1.4–1.5 t m^−3^, respectively [19,20]), which are the most common optical lens materials.

According to the results in Table 1, the optimal level of incorporated plastic waste is around 8%*w*/*w*. Therefore, a series of PWM specimens was prepared, incorporating the waste material as a filler at the optimal level of 8%*w*/*w*, and also above and below this level; then, the compressive strength at 7 days was determined. As mentioned before, specimens of an RM were also prepared and tested for comparison. The mortars’ mix proportions, water-to-cement ratios (A/C), and flow table test results are shown in Table 2 below.

**Figure 8:**
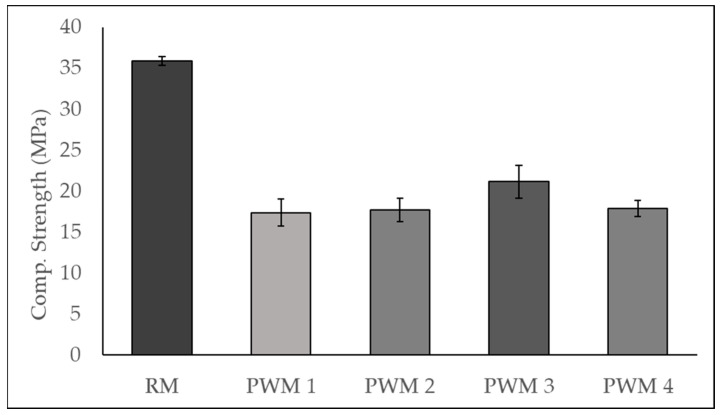
Compressive strength of reference mortar and plastic waste mortars at 7 days.

The A/C was kept constant at 0.48, except for mortar PWM 4, where an additional amount of water had to be added. This was due to the increasing loss of fluidity of the mix with increasing plastic waste content, shown by the decrease in the flow table test diameter. In the case of PWM 4, the A/C had to be slightly adjusted to allow the mortar to have a minimum level of workability. From Figure 8, it can readily be concluded that the substitution of limestone filler with plastic waste in the mortars’ formulation leads to a significant decrease in the compressive strength at 7 days.

### 3.3. Ophthalmic Spectacle Lens Wastewater Valorisation

#### 3.3.1. Microalgae Cultivation and Algal Biomass Characterisation

**Figure 9:**
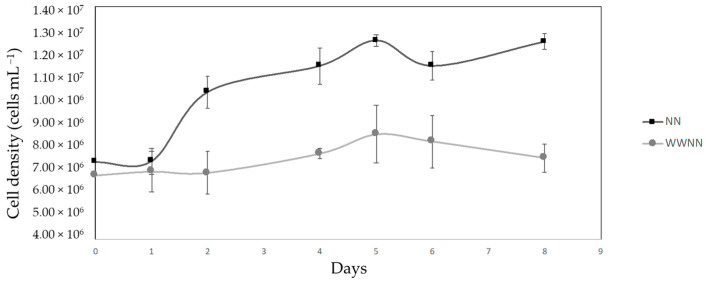
Growth curves of *Nannochloropsis* sp. in continuous cultures. The error bars depict the positive and negative SEM of each data point. The control group is represented by the standard medium for *Nannochloropsis* sp., referred to as f2 medium (NN). This medium did not contain the pollutants present in wastewater.

After separating the suspended solids, the aqueous fraction of the ophthalmic spectacle lens was used as a microalgae culture media. The results in Figure 9 highlight the comparison of the cell growth pattern of *Nannochloropsis* sp. cultivated in the wastewater medium (WWNN) and in the control f2 medium (NN). For one day, the *Nannochloropsis* sp. cells cultivated in industrial wastewater showed identical behaviour to the cells cultivated in the f2 medium. The cells cultivated in the f2 medium displayed a slight but consistent increase in growth; this is a behaviour characteristic of a microalgae population and was observed until the end of the study. It is important to emphasise that the experiments were conducted using cultures initiated with unusually high cell densities. The cells cultivated in the industrial wastewater exhibited evident stability, showing no significant increase or decrease in the presence of the wastewater pollutants; their population remained consistently stable throughout the study. 

A one-way ANOVA was conducted to determine the growth pattern of algae grown in the enriched medium and in wastewater. The results indicate no significant effect, [F = 10.931, *p* = 0.006]. We therefore reject the null hypothesis that different media have varying effects on the growth pattern of *Nannochloropsis* sp.

**Figure 10:**
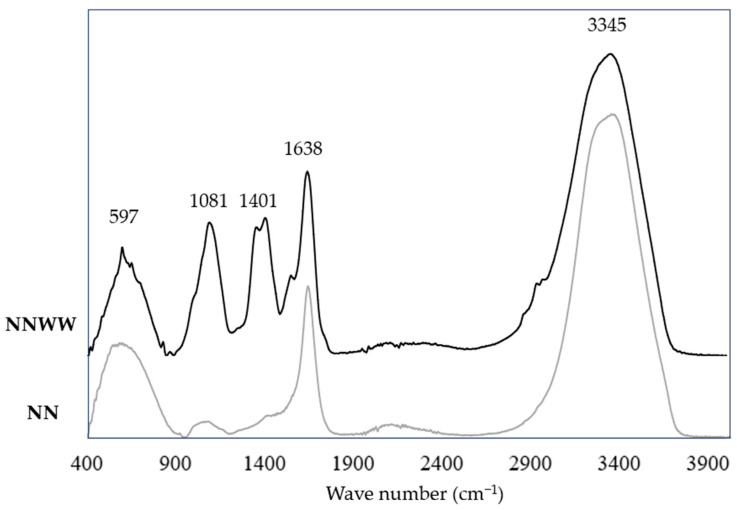
Dried microalgal biomass FTIR-ATR spectra after seven days of growth on f2 medium (NN) and on wastewater (NNWW).

After the cultivation period, the biomass was separated from the aqueous media, dried, and analysed through FTIR-ATR (Figure 10). A first approach showed that the microalgae grown on the standard cultivation media (f2) exhibited strong intensity on the wave numbers 597, 1638, and 3345 cm^−1^, while the microalgae cultivated on the industrial wastewater showed strong intensities on the wave numbers 597, 1081, 1401, 1638, and 3345 cm^−1^. Therefore, this demonstrates that the utilisation of ophthalmic spectacle lens wastewater is suitable for *Nannochloropsis* sp. growth and stimulates the production of bioactive compounds. 

#### 3.3.2. Microalgal Biomass Valorisation Case Study: Ophthalmic Lenses

*Nannochloropsis* sp. is rich in lipids, carbohydrates, and proteins. After the cultivation of *Nannochloropsis* sp. in industrial wastewater, it is possible to obtain feedstock for producing biopolymers. After the extraction of lipids, the lipid-free residues can be neutralised and concentrated into sugars, which can be fermented using a wide range of micro-organisms, such as bacteria, yeasts, and fungi. The product obtained from the fermentation is lactic acid, which is an important biobased block for producing PLA. 

In the context of a circular economy model, we converted ophthalmic spectacle lens waste into ophthalmic spectacle lenses derived from PLA [21] (Figure 2).

**Figure 11:**
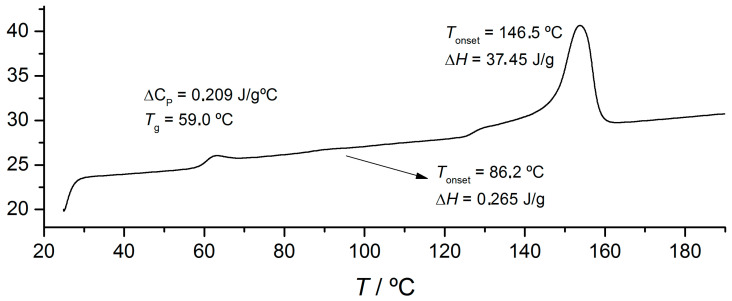
DSC heating curve for PLA. Scanning rate: 10 °C/min.

Feasibility studies on the use of polylactic acid (PLA) in ophthalmic lenses were performed. For comparative purposes, a CR39^®^ polymer lens was used. CR39^®^ is a commercial material that presents very good optical quality. In Table 3, we compare the mechanical properties of two grades of PLA with CR-39^®^. With these, PLA presents better performance when compared with CR-39^®^ and is suitable for application in ophthalmic lenses. The thermal behaviour of PLA (Figure 11) was studied and it was shown that the glass transition temperatures were around 59.0 °C, while the degradation temperatures were observed to be between 140 and 160 °C. The PLTM experiments show devitrification at 63.6 °C, followed by slow crystallisation and finally melting at 163.3 °C. 

The mechanical properties of PLA are similar to those of CR-39^®^ (Table 3).

The refractive index and Abbe number were also measured and showed similar values for the two lenses (Table 4). The refractive index and Abbe numbers of the PLA lenses were 1.46 and 55.24, respectively. 

## 4. Discussion

Mineral glass, polycarbonate, and polyurethanes are some of the polymeric matrixes that can be identified in ophthalmic lens wastewater. A complex system composed of many different types of additives and multicoatings, some of which are considered emerging contaminants, such as BPA, benzene, toluene, and heavy metals, could also be present in this type of effluent [22].

A wide absorption band with a peak at 3332 cm^−1^ suggests the presence of a hydrogen bond in the chemical characterisation of the aqueous portion of industrial wastewater. As a result, the presence of hydrates (H_2_O), hydroxyls (–OH), ammonium, or aminos is confirmed by this band. Because this peak is followed by spectra at frequencies of 1600–1300 and 800–600 cm^−1^, it is possible that hydroxyl compounds are present. A peak at 1635 cm^−1^, for example, shows an unsaturated bond, specifically for double-bond carbon or olefinic compounds (C=C). Conjugations with other double-bond structures, such as C=C, C=O, or aromatic rings, will diminish the intensity frequency of absorption bands that are intense or strong. This also supports the presence of an aromatic ring due to the presence of an absorption band at 1600/1500 cm^−1^ absorption frequency, which is normally linked to a C–H bending vibration with medium to strong absorption intensity; this occasionally has single or multiple absorption bands detected between 850 and 670 cm^−1^. The high intensity at 1650 to 1600 cm^−1^ could be due to double bonds or aromatic molecules [23].

From another perspective, the Raman spectroscopy analysis of the industrial solid residue of the wastewater profile revealed the presence of different polymeric matrices such as polycarbonate (e.g., stretching vibration of the ring appearing at 1605 cm^−1^) [24], polyurethanes (e.g., amide C=O stretching mode band at 1742 cm^−1^ and the N=C=O group symmetric stretching vibration at 1451 cm^−1^) [25], and allyl diglycol carbonate (C=O stretching mode is present in the solid residue spectrum together with the CH_2_ bending vibration at 1451 cm^−1^ and 1288 cm^−1^) [26]. It also showed the presence of persistent organic molecules, such as benzene and toluene, from which the intense band at 1002 cm^−1^ appears, corresponding to the stretching–breathing mode of the aromatic ring [27].

Hence, LDPE and its composites are used in several applications due to their flexibility and mechanical and chemical properties. However, the properties of LDPE composites will depend on several factors, including the properties of the base material and the type of filler material used [28]. Therefore, the spectra of the LDPE and LDPE composites (Figure 6) showed characteristic strong important peaks at 2915 and 2847 cm^−1^ that can be attributed to the asymmetric and symmetric CH_2_ stretching, respectively. These spectra also revealed 1466 and 1376 cm^−1^ bands corresponding to CH_2_ bending deformation and CH_3_ symmetric deformation, respectively [24,27,29]. The peak at 719 cm^−1^ predicts the frequency of the vibration of the rocking mode, which might be due to a disubstitution of cis alkene for ethene, for example. The peak at 1739 cm^−1^ is attributed to C=O stretching, which can suggest cyclic esters of organic acids or an alcohol group and a carboxylic acid. The peak at 1631 cm^−1^ refers to the C–O stretching vibrations of alcohols or vinyl alkyl ethers, and can also be present as a C=C stretch in the structure of aromatic compounds. The peak at 1243 cm^−1^ shows the surface modification or degradation of the polymer chain. Special surface species, such as aliphatic hydrocarbons, are acetates of non-cyclic functional isomers. The peak at 1014 cm^−1^ is characteristic of naturally occurring minerals that are mined from the earth and composed of magnesium, silicon, oxygen, and hydrogen.

Nonetheless, due to the significant decrease in mechanical characteristics observed, the inclusion of wastewater into the polymeric matrix (composites) emerged as a less promising option. However, differing LDPE/residue ratios can increase the mechanical and thermal properties of this composite. Since the solid fraction of the residue could contain different polymeric matrices such as polycarbonate, polyurethanes, and allyl diglycol carbonate, this could influence the mechanical and chemical properties of the final composite. Researchers observed that LDPE has a high interfacial tension with polycarbonate, and that the blends have a phase-separated structure. Polyurethane, on the other hand, is one of the most versatile polymers due to its distinct properties, and can be transformed into sheets, foams, or adhesives.

According to the results presented in Figure 9, by comparing plastic-waste-containing mortars to RM, it can be concluded that the replacement of limestone filler with plastic waste material in proportions ranging from 2.7% to 10.7% of total solids leads to a decrease in the compressive strength of 35% to 45% at 7 days. In this respect, it should be noted that, although the weight proportion of plastic waste is low, the volumetric content is significant, e.g., 8%*w*/*w* corresponds to about 15.5%*v*/*v*. The detrimental effect of the incorporation of plastic waste as a filler on the mechanical properties of cementitious materials has been reported in the literature before [1,2,3,4]. In fact, the compressive strength results obtained may be explained by the poor mechanical properties of the waste material itself (low modulus of elasticity of plastic waste), by its presumable negative impact on the hydration process of cement (restrained cement hydration reaction near the surface of plastic waste), by a low bond strength between the surface of the plastic waste particles and the cement paste, or most probably by a combination of these effects [3,4].

Despite the decrease in compressive strength, plastic-waste-containing mortars still show a performance level that is in line with their use in construction applications. Furthermore, to increase the compatibility between the plastic waste filler and the cementitious matrix and therefore improve the mechanical properties of the final mortar, some pretreatment approaches for the plastic waste may be investigated in the future. The considered pretreatment approaches should include chemical treatments (such as chemical grafting or the use of coupling agents) or physical treatments (such as microwave irradiation or plasma treatment) [5].

On the other hand, focusing only on the plastic-waste-containing mortars, the maximum level of compressive strength was obtained for PWM 3, with 8.0% of plastic waste incorporated, which seems to confirm the suitability of the particle packing optimisation methodology used and the optimal mix design obtained, despite the slight increase in the A/C ratio in PWM4.

The efficiency of microalgae cultivation in wastewater relies on several factors, including cell density, microalgae species, nutrients, molecular structure, and electrostatic interactions between the pollutants [11]. The impact of the wastewater profile on cell density growth is essential to comprehend whether there would be a population decrease when exposed to these factors, as lower cell densities could indicate a crash in the population or an inefficient bioremediation activity, delaying the process and making this method unviable as a complementary option to wastewater treatment. 

It is also important to highlight the convenience of achieving maximum efficiency in bioremediation and the maximum biomass and lipid yields in a relatively short time, especially when considering large-scale production. In industrial and environmental contexts, efficiency is often a critical factor for the sustainability and efficiency of a process. Therefore, the achievement of robust results within a concise timeframe can significantly impact the ecological and economic viability of scaling up the integration of microalgae bioremediation and biobased product production. As such, while our initial experiment spanned 8 days, it reflects the need for a rapid, efficient, and cost-effective approach to bioremediation and biomass production processes.

The results of the bioremediation study presented a notable observation: despite the absence of a conclusive removal of pollutants, *Nannochloropsis* sp. demonstrated cell viability and remarkable resilience when exposed to the wastewater. The fact that the microalgae population remained stable and was sustained when exposed to the wastewater highlights the inherent robustness of *Nannochloropsis*, demonstrated its potential suitability for bioremediation applications, and showed the possibility of utilising ophthalmic spectacle lens wastewater as a culture medium. Furthermore, previous reports [11,12] of *Nannochloropsis* being employed successfully in the bioremediation of various pollutants add an additional layer of optimism to these findings. 

Both growth media (f2 and wastewater) were revealed to be suitable sources for the synthesis of bioactive compounds (Table 5). However, in comparison to the microalgal biomass grown on the standard cultivation growth medium, the characterisation of *Nannochloropsis* sp. grown in wastewater revealed higher intensity peaks at 1081 and 1401 cm^−1^, indicating that this species produces more lipids and polysaccharides under stress conditions. These findings are consistent with the literature, which indicates that different abiotic-stress-induced techniques, such as the presence of a combination of chemical additives and metals, as well as nutritional deprivation, can be employed to increase microalgal lipid and polysaccharide production [29]. 

Furthermore, the value-added product can be reprocessed for a variety of purposes. Microalgae growing in wastewater, for example, can be used to extract bioactive substances if the biomass is safe and of high quality. Indeed, recent research has demonstrated the potential of microalgae wastewater cultivation for the extraction of polyhydroxyalkanoate (PHB), a chemical with numerous therapeutic uses [33,34]. Another bioactive chemical produced by microalgae is chlorophyll, a well-known antioxidant and pigment that can be used as a natural dye in the textile industry [35].

The biomass remaining after lipid extraction still contains carbohydrates, which are equally useful for bioethanol synthesis [36]. The lignin and cellulose content of microalgae can be transformed into methane-rich biogas via anaerobic digestion [37].

Spectacle wearers are frequently advised to undergo an eye exam every two years and, if necessary, update their spectacle prescription, which may require the purchase of a new spectacle frame. As the number of people who use spectacles grows, old spectacles will most certainly end up in landfills. Because spectacle frames will be utilised by a high proportion of the world’s population, developing novel biodegradable spectacle materials is crucial. Hence, the microalgae biomass generated in the bioremediation process can be converted into feedstock for biobased products to develop novel materials for ophthalmic spectacle lens fabrication. In this context, the PLA lens exhibits exceptional mechanical and optical properties, similar to those of CR39. PLA has suitable thermal and mechanical properties that allow it to be processed through various production methods, such as injection moulding, casting, or 3D printing. These properties indicate the significant potential of using the biopolymer PLA derived from microalgae-based bioremediation processes in advanced optical applications and its application in ophthalmic lenses, which can be used in prescription glasses and sunglasses. The fact that PLA, which can be obtained through sustainable and eco-friendly methods and bioremediation processes, can rival the qualities of traditional materials like CR39 opens new possibilities for a greener and more sustainable future for ophthalmic lens production. 

## 5. Conclusions

In this study, we tested the concept of a circular economy approach applied to wastewater from the ophthalmic spectacle lenses industry. In the context of this circular economy proof of concept, our research encompassed the exploration of various potential solutions. Based on the results, it can be concluded that the circular economy model utilising wastewater has the potential to be a sustainable and economically viable solution for waste management and feedstock production. The successful cultivation of microalgae using wastewater as a growth medium not only reduces the environmental impact of wastewater discharge, but also provides a valuable source of biomass that can be used as a feedstock for various applications, including ophthalmic lenses.

This model offers a promising solution for addressing the growing concerns surrounding waste management and the need for sustainable alternatives to conventional materials. Further research is warranted to optimise the efficiency and scalability of the circular economy model. With the approach presented in this work, pollution can be converted into a sustainable economic model. 

This study presents a case study in which industrial wastewater is characterised, reused, and converted. Opportunities and challenges were identified and overcome in the transition towards a more circular economy.

## Figures and Tables

**Table 1:** Volumetric and weight proportions of the mixtures, based on optimised particle packing.

	%*v*/*v*	Density (t/m^3^)	%*w*/*w*
Cement CEM I 42.5 R	27.5%	3.09	33.3%
Sand	57.1%	2.62	58.8%
Plastic waste dust	15.4%	1.30	7.9%

**Table 2:** Series of prepared cementitious mortars. Material proportions are expressed as %*w*/*w*.

	RM	PWM 1	PWM 2	PWM 3	PWM 4
Cement	33.3%	33.3%	33.3%	33.3%	33.3%
Sand	61.3%	64.0%	61.3%	58.7%	56.0%
Limestone filler	5.3%	-	-	-	-
Ophthalmic spectacle lens waste filler	-	2.7%	5.3%	8.0%	10.7%
A/C	0.48	0.48	0.48	0.48	0.50
Flow test diameter (mm)	240	218	190	156	148

**Table 3:** Physical and mechanical properties of PLA and CR39^®^.

Property	PLA	CR-39^®^
Specific weight	1.24	
Notched Izod Impact (ft-lb/in (J/m))	16	0.2–0.4 (15)
Flexural Modulus (kpsi (GPa))	3.6	2.7
Flexural Strength (psi (MPa))	108	91
Tensile Modulus (kpsi (GPa))	3.7	1.6
Tensile Strength at Break (psi (MPa))	57	51

**Table 4:** Refractive indices and Abbe numbers of PLA and CR39.

Polymer	Refractive Index	Abbe Number
PLA	1.46	55.24
CR39	1.50	55.88

**Table 5:** FTIR-ATR peaks for identification of the microalgal biomass grown in the wastewater (NNWW) and on standard growth medium (NN).

Wave Number (cm^−1^)		Bound	Compound	Ref.
NN	NNWW			
3341	3345	*v*(O–H) stretching *v*(N–H) stretching	Water Protein	[30]
1635	1638	Carbonyl (C=O)	Protein	[31,32]
1420	1401	–COOH	Lipids	[30,31,32]
1067	1081	*v*(C–O–C)	Polysaccharides	[30]

## Data Availability

Data are contained within the article.
